# Dr. Linus J. McAdam

**Published:** 1918-05

**Authors:** 


**Dr. Linus J. McAdam,** Barker, N. Y., Niagara University, Medical Department, Buffalo, 1890. Died March 17, age 59. He practiced medicine in Buffalo for many years.

